# Evaluation of the safety and efficacy of a donepezil depot injection in dogs with canine cognitive dysfunction

**DOI:** 10.3389/fvets.2025.1724060

**Published:** 2025-12-15

**Authors:** Min-Hee Kang, Mi-Ae Kang, Hee-Jung Jeon, Ho-chul Shin, Hana Moon, Don-Gil Lee, Hee-Myung Park

**Affiliations:** 1 Department of Bio-Animal Health, Jangan University, Hwaseong, Republic of Korea; 2 Vet & Gene, Seongnam, Republic of Korea; 3 Whanin Pharmaceutical Central Research Institute, Yongin, Republic of Korea; 4 Department of Veterinary Internal Medicine, College of Veterinary Medicine, Konkuk University, Seoul, Republic of Korea

**Keywords:** acetylcholinesterase inhibitor, behavioral assessment, canine cognitive dysfunction, donepezil depot, neurofilament light chain

## Abstract

Canine cognitive dysfunction (CCD) is an age-related neurodegenerative disorder for which effective treatments remain limited, and objective diagnostic and therapeutic assessment tools using biomarkers or neuroimaging are still lacking compared with human Alzheimer’s disease. This study evaluated the safety and efficacy of a long-acting donepezil depot injection in dogs with CCD, using behavioral scores and serum neurofilament light chain (NfL) as primary outcomes, with baseline MRI for diagnostic support. Thirty-two dogs with clinically diagnosed CCD were randomly assigned to a high-dose group (*n* = 11), a low-dose group (*n* = 11), or a control group (*n* = 10). Diagnosis was established based on the Canine Cognitive Dysfunction Rating Scale (CCDR), the CAnine DEmentia Scale (CADES), and DISHAA scoring, and baseline MRI was performed in selected dogs with owner consent. A single intramuscular injection of donepezil depot was administered on day 0, and evaluations were conducted on days 14 and 28. The high-dose group showed significant improvements in CCDR, CADES, and DISHAA at both 14 and 28 days, whereas the low-dose group improved primarily at day 28, with earlier effects limited to CADES (*p* < 0.05). At day 28, both treatment groups had significantly lower serum NfL levels than controls (*p* < 0.05), while within-grou*p* values remained stable. Quality-of-life scores improved in activity, sociability, overall condition, and global QoL. Adverse events were mild and transient. These findings suggest that a single intramuscular injection of long-acting donepezil depot demonstrates favorable safety and potential efficacy in dogs with CCD, with improvements in behavioral scores and NfL supporting its therapeutic potential and highlighting the value of integrating clinical and biomarker-based assessments in future CCD management.

## 1 Introduction

As the life expectancy of companion animals increases, age-associated conditions such as degenerative diseases and neoplasia are reported with greater frequency. Among these, canine cognitive dysfunction (CCD) has emerged as a major geriatric issue because of its progressive neurodegenerative nature and its detrimental impact on the quality of life of affected dogs and their caregivers (1, 2).

CCD typically manifests as progressive cognitive deterioration accompanied by behavioral alterations, which are classified under the acronym DISHAA (disorientation, changes in social interactions, disrupted sleep–wake cycles, house soiling, altered activity levels, and anxiety) (1–3). Comparable to Alzheimer’s disease (AD) in humans, CCD is associated with *β*-amyloid (Aβ) plaque accumulation, tau protein hyperphosphorylation, neuroinflammation, hippocampal atrophy, and gradual loss of cognitive function (2, 4, 5). These pathological and clinical similarities make CCD a valuable naturally occurring model for investigating neurodegenerative processes (4, 5).

The prevalence of CCD increases substantially with age, affecting approximately 8–19% of dogs between 8 and 13 years, 45% of those aged 13–15 years, and up to 67% of dogs 15–17 years old (3). Despite this, CCD is frequently underdiagnosed, as early behavioral changes are often mistaken for normal aging, and the lack of reliable diagnostic biomarkers further delays clinical recognition (3, 6). Neurofilament light chain (NfL) is a neuron-specific structural protein released into blood following axonal damage, and its serum concentration has been proposed as a sensitive biomarker of neurodegeneration in both human and veterinary studies (7–9). Incorporating serum NfL measurement may therefore provide an objective tool for detecting neuronal injury and monitoring disease progression in dogs with CCD. Delayed intervention can lead to progressive cognitive and physical decline, severely affecting the quality of life of both dogs and their caregivers (2, 6).

Management of CCD remains challenging. Current treatment strategies are largely supportive, and only a few regionally approved agents with limited published evidence are available, while no globally recognized veterinary drug specifically targeting CCD has been established (1, 2). Consequently, off-label administration of human AD drugs is common in veterinary practice (1). Donepezil, a selective acetylcholinesterase inhibitor, enhances cholinergic transmission by inhibiting acetylcholine breakdown and has shown significant cognitive benefits in Alzheimer’s disease patients (10). However, its veterinary application remains limited, as clinical data in dogs with CCD are insufficient to determine its efficacy and safety (3, 11).

This study aimed to evaluate the safety and efficacy of a long-acting donepezil depot in dogs with CCD. Two dosage levels were compared to explore potential dose-dependent effects, with behavioral scores, serum NfL, and caregiver-reported quality of life as primary outcomes. To our knowledge, this represents the first controlled clinical trial of a sustained-release donepezil formulation in dogs with CCD.

## 2 Materials and methods

### 2.1 Study design

This study was designed as a multicenter, randomized, parallel-group clinical trial to evaluate the safety and efficacy of a sustained-release donepezil depot in dogs diagnosed with CCD. Three groups were established, including two treatment groups receiving different doses and one control group, with a total observation period of 4 weeks. Dogs were randomly allocated to the three groups at enrollment to minimize potential selection bias. The 4-week observation period was selected based on the expected duration of sustained release from the depot formulation, which was designed to maintain therapeutic plasma concentrations for approximately 1 month. This timeframe allowed for comprehensive assessment of short-term safety and efficacy following a single administration in this pilot study. Safety and efficacy were evaluated using behavioral assessments, serum NfL, and caregiver-reported quality of life. The study was conducted in an open-label format with partial blinding. Owners were informed whether their dogs received the investigational product or served as controls but were unaware of the dose level (high or low). Attending veterinarians were aware of treatment allocation to ensure safety monitoring. To minimize assessment bias, all behavioral questionnaires were completed under veterinary supervision, and serum NfL was measured as an objective biomarker to complement owner-reported outcomes. The open-label design was chosen to reflect practical clinical conditions and to allow comprehensive safety evaluation in this exploratory pilot study. All procedures followed institutional guidelines and were approved by the Institutional Animal Care and Use Committee (approval number: VNG-24-006-1).

### 2.2 Animals and diagnostic criteria

Dogs clinically diagnosed with CCD were recruited from multiple participating veterinary clinics. A total of 32 dogs were enrolled, with 11 dogs assigned to each treatment group and 10 to the control group. The high-dose group received a long-acting donepezil depot at a target dose of approximately 4 mg/kg (calculated as 3.946 mg/kg), the low-dose group received approximately 2 mg/kg (calculated as 1.973 mg/kg), and the control group received no investigational treatment. Group allocation was determined at enrollment following eligibility screening.

Eligible dogs were those showing clinical signs consistent with CCD, confirmed by veterinarian-conducted physical examination and caregiver-reported behavioral assessments using validated scoring systems, including the Canine Cognitive Dysfunction Rating Scale (CCDR), the CAnine DEmentia Scale (CADES), and DISHAA. Dogs were considered to have CCD if at least one of these tools indicated abnormal scores: CCDR ≥50 (12), CADES ≥8 (13), or DISHAA ≥16 (14). MRI findings, when available, were used to support diagnostic confirmation but were not applied as an inclusion criterion. MRI was optional and performed only with owner consent due to ethical and financial constraints. When conducted, MRI provided additional diagnostic reliability and included characteristic structural changes such as hippocampal atrophy, ventricular enlargement, cerebral or cerebellar atrophy, white matter lesions, gray matter signal changes, or midbrain/cerebellar atrophy (2, 15).

Dogs with comorbidities were included only if deemed unlikely to interfere with CCD-related outcomes. Exclusion criteria included systemic illness such as sepsis, poor general condition, severe unrelated diseases, pregnancy, lactation, or participation in another clinical trial. Final inclusion or exclusion decisions were made by the attending veterinarian based on clinical and ethical judgment.

### 2.3 Drug administration

A long-acting donepezil depot was used as the study drug, supplied as a lyophilized powder containing 127.73 mg of donepezil per vial (WhanIn Pharm, Seoul, Korea). The product was stored at 4 °C and reconstituted immediately before administration according to the manufacturer’s instructions. The reconstituted suspension was administered intramuscularly as a single injection on day 0, with the dose volume calculated based on body weight using predetermined dosing tables. These tables were designed to deliver target doses of approximately 2 mg/kg (low-dose) and 4 mg/kg (high-dose); the final calculated values (1.973 and 3.946 mg/kg) reflected the fixed drug concentration after reconstitution.

The selected dose levels were based on previously reported toxicological data from regulatory submissions, which indicated that dogs tolerated single oral doses up to 5 mg/kg without adverse effects and that repeated administration at 1 mg/kg/day for 3 months caused no significant toxicological changes. In addition, preliminary pharmacokinetic data for this intramuscular sustained-release formulation (internal data, on file) showed that systemic exposure at these target doses remained within the safety margins defined by the oral studies, supporting the suitability of the selected dosing regimen. The intramuscular route was chosen for its practicality and for ensuring consistent systemic exposure with this sustained-release formulation.

### 2.4 Efficacy evaluation

Efficacy was evaluated at baseline (D0), mid-study (D14), and study end (D28) using behavioral, biochemical, and caregiver-reported parameters. Cognitive function was assessed using three validated tools: CCDR, CADES, and DISHAA, with structured caregiver questionnaires completed under veterinary guidance at each timepoint. Serum NfL, a marker of axonal injury, was measured at D0, D14, and D28 using a validated immunoassay (NF-light™ ELISA CE-IVD kit; Uman Diagnostics, Quanterix, Umea, Sweden). Quality of life (QoL) was evaluated at D0 and D28 using caregiver-reported questionnaires rating appetite, activity level, sociability, interaction with the owner, and overall condition (7-point Likert scale for individual items and a 0–10 scale for overall QoL).

### 2.5 Safety evaluation

Safety monitoring was performed throughout the 4-week study period by serial physical examinations, vital sign measurements, and laboratory analyses. Body weight, body condition score, rectal temperature, heart rate, respiratory rate, and systolic blood pressure were measured at D0, D14, and D28. Neurological signs related to cholinergic overstimulation (e.g., hypersalivation, tremors, bradycardia, gastrointestinal upset) were closely monitored. Hematological and serum biochemical analyses were performed at D0 and D28, including complete blood count (WBC, LYM, MONO, EOS, RBC, HGB, HCT, PLT) and serum chemistry (ALT, ALP, GGT, TBIL, ALB, GLOB, TP, CHOL, BUN, CREA, PHOS, LIPA, GLU, Ca, K^+^, Na^+^, Cl^−^), to evaluate hepatic, renal, and metabolic safety. All clinical signs observed during the study were recorded by at-tending veterinarians and caregivers, and their causal relationship to the investigational product was assessed by the investigators. Although attending veterinarians were aware of treatment allocation to ensure appropriate safety monitoring, potential observer bias was minimized by applying uniform examination criteria and incorporating objective laboratory and biomarker data into the safety evaluation.

### 2.6 Statistical analysis

All data were expressed as mean ± standard deviation (SD) or mean ± standard error (SE). Missing data from one dog in the low-dose group (died after D14) were imputed using mean substitution for repeated measures, whereas this case was excluded from quality-of-life analysis, which was assessed only at D0 and D28. Outliers were identified by Tukey’s rule (1.5 × IQR) using box plots, resulting in the exclusion of one high-dose dog from CADES and DISHAA analyses. To account for inter-individual baseline variability, relative ratios were calculated for behavioral scores and NfL levels. Statistical comparisons were performed using two-way repeated measures ANOVA to evaluate time, group, and time × group effects, with Greenhouse–Geisser correction applied when sphericity was violated. *Post-hoc* analyses were conducted using Dunnett’s or Bonferroni tests, and adverse event frequencies were compared using Pear-son’s chi-square test. Statistical significance was set at *p* < 0.05, and all analyses were performed using GraphPad Prism (version 9.5.1; GraphPad Software Inc., Boston, MA, USA).

## 3 Results

### 3.1 Baseline characteristics of enrolled dogs

A total of 32 dogs were enrolled and allocated into three groups: 11 dogs in the high-dose group, 11 dogs in the low-dose group, and 10 dogs in the control group. One dog in the low-dose group (17 years old) died of acute pneumonia after day 14, which was judged unrelated to the investigational drug. The mean age was similar between treatment groups (15.12 ± 1.42 years in the high-dose group; 15.51 ± 1.40 years in the low-dose group), whereas the control group was slightly younger (13.11 ± 1.04 years). The sex distribution was comparable, with a predominance of neutered animals. Small-breed dogs, particularly Poodles, Maltese, and Shih Tzus, were the most frequently represented (Table 1).

**Table 1:** Demographic characteristics of enrolled dogs.

Group (*n*)	Age (years, mean ± SD)	Gender composition	Representative breeds (*n*)
Group 1 (11)	15.12 ± 1.42	Male: 4, female: 8, neutered male/female: 20	Poodle (12), Maltese (8), Shih Tzu (4), Chihuahua (3), Mix (3), Cocker Spaniel (2)
Group 2 (11)	15.51 ± 1.40		
Control (10)	13.11 ± 1.04		

*n* = number of animals; Group 1 = high-dose group; Group 2 = low-dose group.

Baseline MRI was performed in 21 dogs (7 in the high-dose group, 5 in the low-dose group, and 9 in the control group) with owner consent. CCD-associated structural changes were observed in 57% (4/7) of the high-dose group, 40% (2/5) of the low-dose group, and 44% (4/9) of the control group, with no significant intergroup differences (*p* > 0.05). The most common abnormalities included cerebral atrophy, ventricular enlargement, and periventricular signal changes, which were similarly distributed across groups, suggesting comparable baseline structural brain status.

### 3.2 Behavioral outcomes

**Figure 1:**
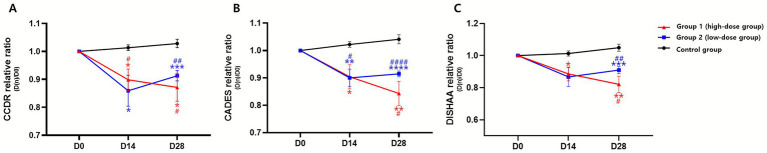
Changes in behavioral assessment scores following administration of donepezil depot (WIF-2401) in dogs with cognitive dysfunction (CCD). **(A)** Canine Cognitive Dysfunction Rating (CCDR) relative ratio. **(B)** Cognitive Dysfunction Syndrome Assessment for Dogs (CADES) relative ratio, and **(C)** DISHAA relative ratio were evaluated at baseline (D0), 14 days (D14), and 28 days (D28) after treatment. Scores are expressed as the relative ratio to baseline (D[*n*]/D0). Data are presented as mean ± SEM. *p < 0.05, **p < 0.01, ***p < 0.001, ***p < 0.0001 vs. D0 (within groups); #p < 0.05, ##p < 0.01, ###p < 0.001, ####p < 0.0001 vs. control (between groups at the same time point).

Behavioral changes were evaluated using the CCDR, CADES, and DISHAA scales at baseline, D14, and D28. The same three questionnaires were administered at each evaluation point for every dog to ensure consistency in longitudinal assessment. The results are summarized in Table 2 and illustrated in Figure 1.

**Table 2:** Behavioral assessment scores (CCDR, CADES, and DISHAA) in dogs with canine cognitive dysfunction following donepezil depot administration.

Scale			Group 1 (*n* = 11)		Group 2 (*n* = 11)		Control (*n* = 10)	
			Mean ± SD	Within group (*p*)	Mean ± SD	Within group (*p*)	Mean ± SD	Within group (*p*)
CCDR	D0	Score	46.64 ± 9.91	–	49.55 ± 6.76	–	43.80 ± 5.29	–
		Relative ratio	1.00 ± 0.00		1.00 ± 0.00		1.00 ± 0.00	
	D14	Score	41.55 ± 9.41	<0.05	41.82 ± 7.91	–	44.30 ± 5.03	–
		Relative ratio	0.90 ± 0.13		0.86 ± 0.18		1.01 ± 0.03	
		Between group (*p*)	<0.05		<0.05		–	
	D28	Score	40.73 ± 12.14	<0.05	45.14 ± 6.63	<0.01	44.90 ± 5.04	–
		Relative ratio	0.87 ± 0.16		0.91 ± 0.06		1.03 ± 0.05	
		Between group (*p*)	<0.05		<0.001		–	
		Source	T		G		T × G	
		*F*-value	7.99		5.40		3.83	
		*p*-value	<0.01		<0.05		<0.01	

Scale			Group 1 (*n* = 10)		Group 2 (*n* = 11)		Control (*n* = 10)	
			Mean ± SD	Within group (*p*)	Mean ± SD	Within group (*p*)	Mean ± SD	Within group (*p*)
CADES	D0	Score	54.70 ± 4.54	–	64.82 ±16.77	–	47.80 ± 9.03	–
		Relative ratio	1.00 ± 0.00		1.00 ± 0.00		1.00 ± 0.00	
	D14	Score	47.70 ± 0.37	–	57.73 ±14.47	<0.05	49.00 ± 0.00	–
		Relative ratio	0.90 ± 0.14		0.90 ± 0.11		1.02 ± 0.03	
		Between group (*p*)	<0.05		<0.01		–	
	D28	Score	45.60 ± 2.79	<0.05	59.50 ± 6.37	<0.0001	49.70 ± 9.23	–
		Relative ratio	0.84 ± 0.14		0.92 ± 0.03		1.04 ± 0.05	
		Between group (*p*)	<0.01		<0.0001		-	
		Source	T		G		T × G	
		*F*-value	10.26		9.07		7.09	
		*p-*value	<0.001		<0.001		<0.001	

Scale			Group 1 (*n* = 10)		Group 2 (*n* = 11)		Control (*n* = 10)	
			Mean ± SD	Within group (*p*)	Mean ± SD	Within group (*p*)	Mean ± SD	Within group (*p*)
DISHAA	D0	Score	30.80 ± 10.27	–	35.55 ± 7.47	–	29.70 ± 4.45	–
		Relative ratio	1.00 ± 0.00		1.00 ± 0.00		1.00 ± 0.00	
	D14	Score	26.70 ± 8.30	–	30.18 ± 7.65	–	30.00 ± 4.03	–
		Relative ratio	0.89 ± 0.14		0.87 ± 0.19		1.01 ± 0.05	
		Between group (*p*)	<0.05		–		–	
	D28	Score	25.20 ± 10.30	<0.05	32.36 ± 7.78	<0.01	31.10 ± 4.89	–
		Relative ratio	0.82 ± 0.16		0.91 ± 0.07		1.05 ± 0.07	
		Between group (*p*)	<0.01		<0.001		–	
		Source	T		G		T × G	
		*F*-value	7.54		7.14		4.96	
		*p-*value	<0.01		<0.01		<0.01	

*n* = number of animals; Group 1 = high-dose group; Group 2 = low-dose group; D = study days (D0 = baseline, D14 = day 14, D28 = day 28); *T* = time effect; *G* = group effect; Mean ± SD = mean and standard deviation; Relative ratio = post-treatment score divided by baseline (D0, set as 1.00); Within-group (*p*) = *p*-value for comparisons with baseline within each group (only *p* < 0.05 shown); Between-group (*p*) = *p*-value for comparisons between each treatment group and the control at the same time point (only *p* < 0.05 shown).

CCDR (Canine Cognitive Dysfunction Rating scale), CADES (CAnine DEmentia Scale), and DISHAA (disorientation, altered social interactions, disrupted sleep–wake cycles, house soiling, altered activity levels, and anxiety) are validated behavioral assessment tools for canine cognitive dysfunction. Higher scores indicate greater cognitive impairment.

At baseline, scores were comparable across groups for all three scales. When ex-pressed as relative ratios to baseline (D0 = 1.00), both treatment groups showed statistically significant improvements compared with the control. In the CCDR scale, the high-dose group exhibited notable but moderate reductions at D14 and D28 (both *p* < 0.05), while the low-dose group showed a measurable decrease only at D28 (*p* < 0.01). For CADES, the high-dose group demonstrated a significant reduction at D28 (*p* < 0.05), and the low-dose group showed consistent decreases at both D14 (*p* < 0.05) and D28 (*p* < 0.0001). In the DISHAA scale, the high-dose group showed significant reductions at D28 (*p* < 0.05), whereas the low-dose group also exhibited a significant decrease at D28 (*p* < 0.01), with no significant changes at D14. In contrast, the control group showed no significant changes over time in any of the scales.

Between-group comparisons further confirmed that the high-dose group had lower relative ratios than the control group at both D14 and D28 across all three scales (*p* < 0.05 to *p* < 0.01). The low-dose group also showed significantly lower values than the control, at D14 for CCDR (*p* < 0.05) and CADES (*p* < 0.01), and at D28 for all three scales (*p* < 0.01 to *p* < 0.0001). Significant main effects of time, group, and their interaction were consistently observed across the three behavioral scales (*p* < 0.05 to *p* < 0.001).

These findings indicate that donepezil depot administration resulted in statistically significant and measurable improvements in behavioral scores in treated groups compared with controls, with the magnitude and timing of changes differing across assessment tools.

### 3.3 Serum NfL levels

Serum NfL concentrations were measured as a biomarker of axonal injury. At baseline, levels were comparable across groups. When expressed as relative ratios to baseline (D0 = 1.00), the high-dose group remained close to baseline over time (0.95 ± 0.11 at D14; 0.94 ± 0.13 at D28; not significant vs. baseline). The low-dose group showed a decrease at D14 (0.86 ± 0.22) with a partial rebound at D28 (0.92 ± 0.29); neither time point differed significantly from baseline. In contrast, the control group exhibited a progressive rise, reaching 1.52 ± 0.43 at D28 (*p* < 0.01 vs. baseline).

**Figure 2:**
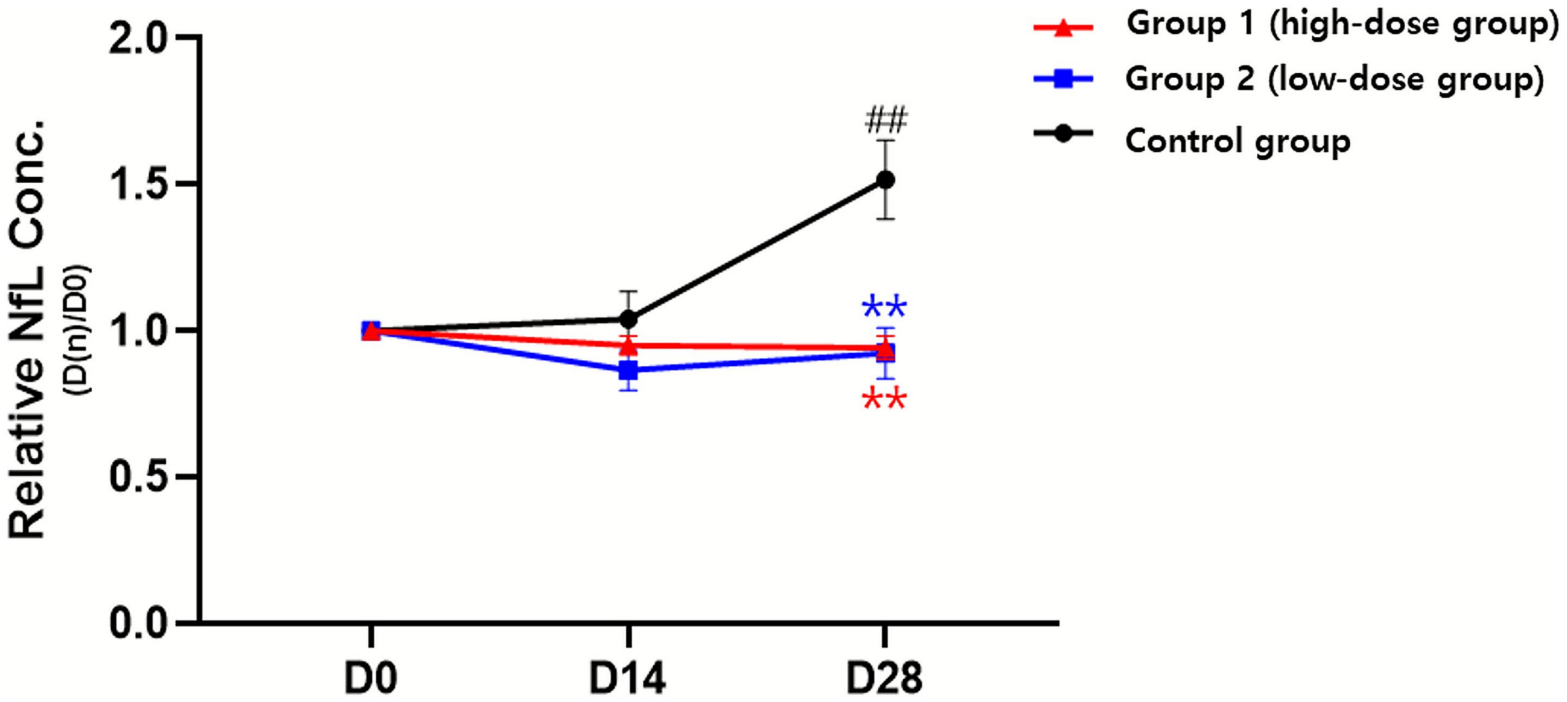
Changes in serum neurofilament light chain (NfL) concentration following administration of donepezil depot in dogs with cognitive dysfunction (CCD). Relative NfL concentrations (D[n]/D0) were evaluated at baseline (D0), 14 days (D14), and 28 days (D28) after treatment. Data are presented as mean ± SEM. *p < 0.05, **p < 0.01 vs. D0 (within group); #*p* < 0.05, ##*p* < 0.01 vs. control (between groups at the same time point).

Between-group comparisons at D28 showed that both treatment groups had significantly lower relative NfL ratios than the control (both *p* < 0.01). Analysis of variance demonstrated significant main effects of time (*p* < 0.01), group (*p* < 0.001), and time- group interaction (*p* < 0.0001) (Table 3 and Figure 2).

**Table 3:** Serum neurofilament light chain (NfL) concentrations and relative ratios in dogs with canine cognitive dysfunction following donepezil depot administration.

		Group 1 (*n* = 11)		Group 2 (*n* = 11)		Control (*n* = 10)	
		Mean ± SD	Within group (*p*)	Mean ± SD	Within group (*p*)	Mean ± SD	Within group (*p*)
D0	Concentration	266.04 ± 156.99	–	244.72 ± 211.24	–	218.86 ± 145.82	–
	Relative ratio	1.00 ± 0.00		1.00 ± 0.00		1.00 ± 0.00	
D14	Concentration	254.79 ± 160.88	–	202.99 ± 167.67	–	205.29 ± 123.91	–
	Relative ratio	0.95 ± 0.11		0.86 ± 0.22		1.04 ± 0.30	
	Between group (*p*)	–		–		–	
D28	Concentration	251.98 ± 156.49	–	228.96 ± 201.23	–	343.44 ± 289.07	<0.01
	Relative ratio	0.94 ± 0.13		0.92 ± 0.29		1.52 ± 0.43	
	Between group (*p*)	<0.01		<0.01		–	
	Source	T		G		T × G	
	*F*-value	7.05		9.17		8.79	
	*p-*value	<0.01		<0.001		<0.0001	

*n* = number of animals; Group 1 = high-dose group; Group 2 = low-dose group; *D* = study days (D0 = baseline, D14 = day 14, D28 = day 28); *T* = time effect; *G* = group effect; Mean ± SD = mean and standard deviation; Relative ratio = post-treatment NfL concentration divided by baseline (D0, set as 1.00); Within-group (*p*) = *p*-value for statistical comparisons with baseline within each group (only *p* < 0.05 shown); Between-group (*p*) = *p*-value for comparisons between each treatment group and the control at the same time point (only *p* < 0.05 shown).

These findings indicate different NfL trends, showing stability in the treatment groups but a marked increase in the control group, suggesting that donepezil depot may mitigate NfL increases associated with disease progression.

### 3.4 Owner-reported QoL

**Figure 3:**
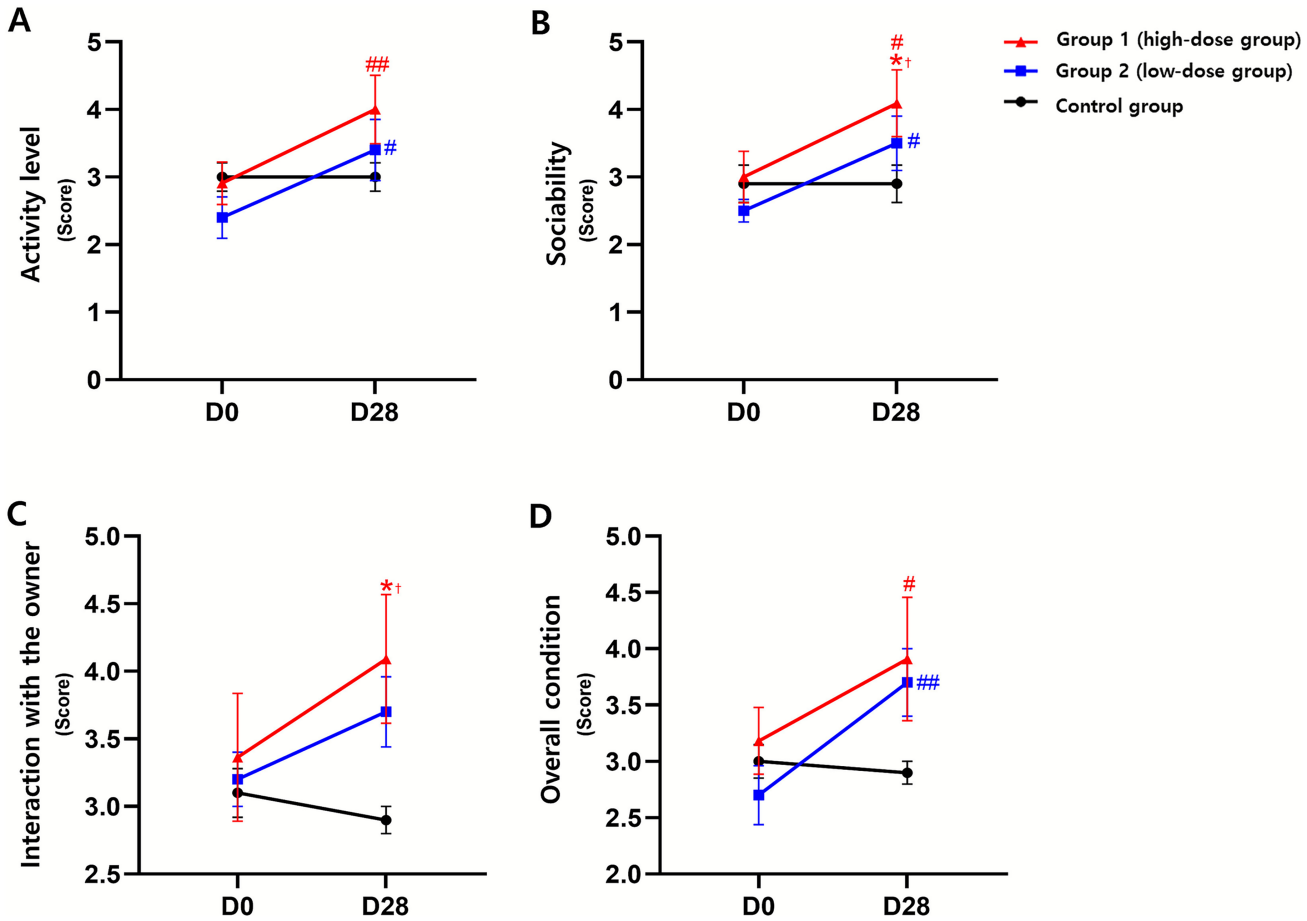
Changes in owner-reported quality-of-life (QoL) scores following administration of donepezil depot in dogs with cognitive dysfunction (CCD). **(A)** Activity level, **(B)** sociability, **(C)** Interaction with the owner, and **(D)** overall condition were assessed using an owner-completed questionnaire at baseline (D0) and 28 days (D28) after treatment. Data are presented as mean ± SEM. *p < 0.05, *p < 0.01 vs. D0 (within group); #*p* < 0.05, ##*p* < 0.01 vs. control (between groups at the same time point); †exploratory interpretation, indicating statistical significance observed in *post-hoc* analysis without a predefined hypothesis.

QoL was assessed at baseline and D28 using an owner-completed questionnaire that evaluated appetite, activity level, sociability, interaction with the owner, overall condition, and overall QoL. Results are summarized in Figure 3, with full data provided in Supplementary Table 1.

Appetite showed a slight improvement trend across groups, but no significant effects of time, group, or time–group interaction were detected. Activity level demonstrated a significant time effect (*p* < 0.001) and a time–group interaction (*p* < 0.05). The high-dose group increased from 2.91 ± 1.04 to 4.00 ± 1.67 (*p* < 0.01 vs. D0), and the low-dose group from 2.40 ± 0.97 to 3.40 ± 1.43 (*p* < 0.05 vs. D0), whereas the control remained unchanged (3.00 ± 0.67 at both time points). Sociability showed a significant time effect (*p* < 0.01); scores increased in the high-dose group from 3.00 ± 1.26 to 4.09 ± 1.64 (*p* < 0.05 vs. D0) and in the low-dose group from 2.50 ± 0.53 to 3.50 ± 1.27 (*p* < 0.05 vs. D0), while the control showed no change. Exploratory *post hoc* comparisons indicated a higher D28 score in the high-dose group versus control (*p* < 0.05). Interaction with the owner showed no significant main effects; however, exploratory post hoc analysis revealed a higher D28 score in the high-dose group compared with control (4.09 ± 1.58 vs. 2.90 ± 0.32, *p* < 0.05). Overall condition demonstrated significant effects of time (*p* < 0.01) and time–group interaction (*p* < 0.05). Scores increased from 3.18 ± 0.98 to 3.91 ± 1.81 in the high-dose group (*p* < 0.05 vs. D0) and from 2.70 ± 0.82 to 3.70 ± 0.95 in the low-dose group (*p* < 0.01 vs. D0), whereas the control remained unchanged. Exploratory *post hoc* comparisons at D28 indicated higher scores in treated groups than in the control (*p* < 0.05 to *p* < 0.01).

Overall QoL (0–10 scale) improved over time (*p* < 0.05), increasing from 4.27 ± 2.28 to 5.36 ± 2.80 in the high-dose group (*p* < 0.05 vs. D0) and from 2.90 ± 1.10 to 4.50 ± 1.96 in the low-dose group (*p* < 0.01 vs. D0), while the control showed no change. An exploratory post hoc comparison indicated a higher D28 score in the high-dose group than in the control (*p* < 0.05).

Together, activity level, sociability, overall condition, and overall QoL showed statistically significant improvements within the treatment groups, whereas the control remained unchanged. However, these outcomes were based on caregiver-reported data and should be interpreted as supportive observations consistent with behavioral and biomarker findings, rather than definitive evidence of treatment efficacy (Figure 3, Supplementary Table 1).

### 3.5 Safety evaluation

Serial evaluations of physical examinations, vital signs, and laboratory parameters showed no clinically relevant abnormalities attributable to donepezil depot administration during the 4-week study period (Supplementary Tables 2–4).

All vital signs, including BW, temperature, HR, RR, and SBP, remained within physiologic reference ranges. BCS showed a mild decrease over time across all groups (*p* < 0.01), but this change was not treatment-related. SBP differed slightly between groups (*p* < 0.05) due to baseline variability, but absolute values were consistently within normal clinical limits.

Hematologic parameters showed only minor temporal variations in LYM, RBC, HGB, and HCT (all *p* < 0.05–0.01, time effect), which were transient and remained within reference ranges across groups. No significant group or interaction effects were observed, and no clinically meaningful hematologic abnormalities occurred. Serum biochemistry values were stable across groups, with all parameters remaining within reference ranges. Albumin showed a statistical difference by time, group, and time–group interaction (all *p* < 0.05), attributable to mild variations in the control group; however, values stayed within reference intervals and were not considered treatment-related. No other liver, renal, or metabolic indicators showed significant or clinically relevant alterations.

Overall, these findings suggest that both low- and high-dose donepezil depot were well tolerated, with no clinically relevant abnormalities detected in physical, hematologic, or biochemical evaluations during the 4-week study period.

### 3.6 Adverse events

Adverse events were monitored and assessed throughout the 4-week study by attending veterinarians and caregivers. The overall frequency and distribution of clinical signs are summarized in Supplementary Table 5, and the detailed causality assessments are provided in Supplementary Table 6.

The incidence of adverse events was low across all groups, with no significant differences between treatment and control groups. Most observed clinical signs, including weight loss, lethargy, decreased appetite, polyuria, alopecia, vomiting, and diarrhea, were either present prior to treatment or commonly associated with aging in geriatric dogs.

In the high-dose group, one dog developed transient erythema of the inner ear and mild lethargy at D14, which resolved spontaneously without intervention. Another dog in the same group experienced diarrhea at D28, accompanied by mild weight loss and polyuria; however, the delayed onset of 3 weeks post-treatment and spontaneous resolution led to its evaluation as unrelated treatment. In the low-dose group, one dog died of acute pneumonia at D28, which the attending veterinarian attributed to advanced age rather than drug administration.

Overall, no treatment-related adverse events were identified, indicating that donepezil depot was well tolerated during the study period.

## 4 Discussion

This clinical trial evaluated the safety and efficacy of a single intramuscular injection of a sustained-release donepezil depot in dogs with CCD. Both high- and low-dose groups demonstrated measurable cognitive improvements, although the timing and magnitude varied across behavioral scales. For example, earlier effects were observed in the low-dose group for CADES, while the high-dose group showed earlier and sustained improvement in CCDR. Taken together, the three scales consistently indicated benefits of treatment compared with controls. Serum NfL levels, a biomarker of axonal injury, remained stable in treated dogs, whereas levels increased in controls, suggesting a potential neuroprotective effect. QoL scores also improved, particularly in activity, sociability, and overall condition, and no serious treatment-related adverse events were identified.

In veterinary practice, treatment of CCD remains largely symptomatic and supportive, with few pharmacologic interventions formally evaluated in controlled clinical trials (1). Most therapeutic approaches have been extrapolated from human AD, where cholinergic dysfunction is a well-established pathomechanism (16). Donepezil and other cholinesterase inhibitors (ChEIs) have consistently improved cognitive performance in AD by enhancing cholinergic transmission and synaptic plasticity (10, 16). These mechanistic insights provide a rationale for adopting similar strategies in CCD, which shares pathological features with AD, such as Aβ deposition, tau hyperphosphorylation, and cholinergic deficits (2, 3).

Only a few controlled studies have evaluated ChEIs in dogs. In one study, aged Beagle dogs receiving oral donepezil showed improved delayed non-matching-to-position (DNMP) task performance, with optimal effects at 1.5 mg/kg, supporting the role of cholinergic enhancement in mitigating cognitive decline (17). However, higher doses (6 mg/kg) induced cholinergic adverse effects such as vomiting, hypersalivation, and tremors, indicating a narrow therapeutic window for oral regimens. More recently, a butyrylcholinesterase inhibitor (BChEi) improved cognitive function and quality of life in moderately affected client-owned dogs assessed with CADES and cognitive performance tests; however, gastrointestinal adverse events frequently led to treatment discontinuation in severely affected cases (18). These findings highlight both the therapeutic potential and safety limitations of ChEIs in CCD management, emphasizing the need for alternative delivery strategies that maintain effective systemic exposure while reducing dose-dependent adverse effects. The long-acting donepezil depot used in this study was designed to address these limitations and, importantly, enabled evaluation with both behavioral outcomes and the objective biomarker NfL.

Cholinergic enhancement is thought to exert neuroprotective effects not only through symptomatic improvement but also by attenuating neuroinflammation and axonal injury, as demonstrated in experimental models of neurodegeneration (19, 20). Serum NfL is a sensitive biomarker of axonal injury, with increased levels linked to neurodegeneration and disease progression in CCD and other neurodegenerative disorders (7–9). In this study, both treatment groups showed stable or slightly reduced NfL levels, whereas controls exhibited a progressive increase, suggesting that sustained cholinergic modulation achieved with the depot formulation may help limit ongoing axonal damage in CCD. The earlier and more consistent behavioral improvements in the high-dose group support a possible dose-dependent response, which has been difficult to investigate with oral regimens due to their narrow therapeutic window (17). The parallel trends between behavioral scores and NfL changes in this study provide preliminary evidence that clinical improvement may reflect not only functional compensation but also potential disease-modifying effects, warranting further investigation in larger, long-term studies.

The favorable safety profile and single-dose administration of the donepezil depot formulation offer practical advantages for clinical use, particularly in geriatric dogs where repeated oral dosing may be challenging due to poor compliance or coexisting medical conditions. Sustained drug exposure achieved with this formulation may help maintain therapeutic cholinergic modulation while reducing the risk of dose-related adverse effects that limit the use of oral ChEIs. In addition, improvements in activity, sociability, and overall quality of life reported by caregivers highlight its potential to provide meaningful clinical benefits not only for affected dogs but also for their owners, who often experience significant emotional and caregiving burdens. These findings suggest that the depot formulation could represent a valuable addition to current management strategies for CCD, especially in cases requiring long-term maintenance therapy.

Despite these encouraging results, several limitations should be acknowledged. The relatively short observation period precludes conclusions about the long-term durability of cognitive and neuroprotective effects. Although NfL reduction suggests attenuation of axonal injury, whether this reflects structural preservation of the brain remains uncertain, as MRI was performed only in a subset of dogs due to ethical and financial constraints. This limited imaging dataset also prevented robust correlation analyses between structural changes, behavioral improvements, and biomarker dynamics. Additionally, most enrolled dogs had mild to moderate CCD, leaving the safety and efficacy profile in severe cases undetermined. Furthermore, while serum NfL measurement by ELISA is a widely accepted and practical approach in veterinary research, its analytical sensitivity is lower than that of ultra-sensitive assays such as the single molecule array, which may limit the detection of subtle biomarker fluctuations. Finally, although standardized behavioral scoring systems were used, caregiver-reported outcomes remain subject to inherent subjectivity. A potential placebo influence cannot be completely excluded despite veterinary supervision during assessments. Owner-reported assessments can show placebo-associated improvement, and recent evidence indicates that the magnitude of this effect varies depending on the instrument used. CADES has been shown to exhibit a strong and persistent placebo response, with effect sizes in the placebo arm of a randomized trial remaining elevated over several months (Hedges’ g up to 0.76), whereas CCDR demonstrates only minimal and short-lived placebo-related change (21). Objective cognitive tests, in contrast, show no measurable placebo influence. These findings suggest that some degree of nonspecific caregiver-driven improvement may occur in questionnaire-based assessments. The use of multiple complementary behavioral tools in the present study, together with the inclusion of an objective biomarker such as serum NfL, helps mitigate this limitation and provides a more balanced evaluation of treatment response. These considerations also apply to the owner-reported QoL evaluation, which should be interpreted as supportive rather than confirmatory evidence of treatment benefit. These factors highlight the need for more objective endpoints, such as advanced neuroimaging or electrophysiological assessments, in future trials. In addition, this pilot trial was conducted with a limited sample size, and a formal power analysis was not performed; therefore, the study may not have been sufficiently powered to detect smaller treatment effects. This limitation should be considered when interpreting the results, and future large-scale studies with appropriate statistical power are warranted.

Future studies should extend follow-up periods to clarify the persistence of cognitive and structural benefits. Incorporating serial MRI or positron emission tomography could help evaluate amyloid or tau pathology and confirm structural brain preservation. Evaluating dogs with severe CCD will help define stage-specific therapeutic responses and safety considerations. Moreover, integrating additional biomarkers, including glial fibrillary acidic protein (GFAP) and other neurodegeneration-related markers, may enhance early detection of treatment response and guide individualized therapeutic strategies. Investigating combination therapies that target oxidative stress or neuroinflammation could further optimize clinical outcomes.

This study provides preliminary evidence that a single intramuscular injection of a sustained-release donepezil depot can improve cognitive function in dogs with CCD, with good tolerability and potential neuroprotective effects supported by NfL stabilization. Integrating behavioral assessments with biomarker analysis provides a practical and objective framework for evaluating therapeutic efficacy. The long-acting depot formulation also addresses compliance challenges in geriatric dogs and may represent a clinically relevant option for long-term management of CCD. Confirmation in larger, placebo-controlled, and extended studies is warranted.

## Data Availability

The original contributions presented in the study are included in the article/Supplementary material, further inquiries can be directed to the corresponding author.
