# Supplementary material for: Evaluation of the safety and efficacy of a donepezil depot injection in dogs with canine cognitive dysfunction
Source: Front Vet Sci. 2025 Dec 15;12:1724060. doi: 10.3389/fvets.2025.1724060 (PMC12745245; doi:10.3389/fvets.2025.1724060)
Supplement: Supplementary file 1 [file Table_1.DOCX]

**Supplementary Table 1.** Detailed caregiver-reported quality of life (QoL) scores, including individual items (appetite, activity level, sociability, interaction with the owner, and overall condition) and overall QoL in dogs with canine cognitive dysfunction

| **Evaluation parameters**  **(reference range)** |  | | | **Group 1 (n=11)** | | | **Group 2 (n=11)** | | | **Control (n=10)** | | |
| --- | --- | --- | --- | --- | --- | --- | --- | --- | --- | --- | --- | --- |
|  |  |  |  | **Mean ± SD** | | **Within group (p)** | **Mean ± SD** | | **Within group (p)** | **Mean ± SD** | | **Within group (p)** |
| Appetite  (1–7 score) | D0 | Score | | 3.36 ± 0.81 | | – | 3.50 ± 0.71 | | – | 3.20 ± 0.63 | | – |
|  |  | Between group (*p*) | | – | | | – | | | – | | |
|  | D28 | Score | | 3.73 ± 1.85 | | – | 3.80 ± 1.03 | | – | 3.10 ± 0.57 | | – |
|  |  | Between group (*p*) | | – | | | – | | | – | | |
|  | **Source** | | | T | | | G | | | T × G | | |
|  | **F value** | | | 1.11 | | | 0.82 | | | 0.66 | | |
|  | ***p* value** | | | – | | | – | | | – | | |
| **Evaluation parameters (reference range)** |  | | | **Group 1 (*n*=11)** | | | **Group 2 (*n*=11)** | | | **Control (*n*=10)** | | |
|  |  |  |  | **Mean ± SD** | **Within group (*p*)** | | **Mean ± SD** | **Within group (*p*)** | | **Mean ± SD** | **Within group (*p*)** | |
| Activity level  (1–7 score) | D0 | | Score | 2.91 ± 1.04 | – | | 2.40 ± 0.97 | – | | 3.00 ±0.67 | – | |
|  |  |  | Between group (*p*) | – | | | – | | | – | | |
|  | D28 | | Score | 4.00 ±1.67 | <0.01 | | 3.40 ±1.43 | <0.05 | | 3.00 ±0.67 | - | |
|  |  |  | Between group (*p*) | – | | | – | | | – | | |
|  | **Source** | | | T | | | G | | | T × G | | |
|  | ***F* value** | | | 13.61 | | | 0.89 | | | 3.39 | | |
|  | ***p* value** | | | <0.001 | | | – | | | <0.05 | | |
| **Evaluation parameters (reference range)** |  | | | **Group 1 (*n*=11)** | | | **Group 2 (*n*=11)** | | | **Control (*n*=10)** | | |
|  |  |  |  | **Mean ± SD** | **Within group (*p*)** | | **Mean ± SD** | **Within group (*p*)** | | **Mean ± SD** | **Within group (*p*)** | |
| Sociability  (1–7 score) | D0 | | Score | 3.00 ± 1.26 | – | | 2.50 ± 0.53 | – | | 2.90 ± 0.88 | – | |
|  |  |  | Between group (*p*) | – | | | – | | | – | | |
|  | D28 | | Score | 4.09 ± 1.64 | <0.05 | | 3.50 ± 1.27 | <0.05 | | 2.90 ± 0.88 | - | |
|  |  |  | Between group (*p*) | <0.05 | | | – | | | – | | |
|  | **Source** | | | T | | | G | | | T × G | | |
|  | ***F* value** | | | 9.81 | | | 1.38 | | | 2.44 | | |
|  | ***p* value** | | | <0.01 | | | – | | | – | | |
| **Evaluation parameters (reference range)** |  | | | **Group 1 (*n*=11)** | | | **Group 2 (*n*=11)** | | | **Control (*n*=10)** | | |
|  |  |  |  | **Mean ± SD** | **Within group (*p*)** | | **Mean ± SD** | **Within group (*p*)** | | **Mean ± SD** | **Within group (*p*)** | |
| Interaction with the owner  (1–7 score) | D0 | | Score | 3.36 ± 1.57 | – | | 3.20 ± 0.63 | – | | 3.10 ± 0.57 | – | |
|  |  |  | Between group (*p*) | – | | | – | | | – | | |
|  | D28 | | Score | 4.09 ± 1.58 | – | | 3.70 ± 0.82 | – | | 2.90 ± 0.32 | – | |
|  |  |  | Between group (*p*) | <0.05 | | | – | | | – | | |
|  | **Source** | | | T | | | G | | | T × G | | |
|  | **F value** | | | 2.20 | | | 1.97 | | | 1.46 | | |
|  | ***p* value** | | | – | | | – | | | – | | |
| **Evaluation parameters (reference range)** |  | | | **Group 1 (*n*=11)** | | | **Group 2 (*n*=11)** | | | **Control (*n*=10)** | | |
|  |  |  |  | **Mean ± SD** | **Within group (*p*)** | | **Mean ± SD** | **Within group (*p*)** | | **Mean ± SD** | **Within group (*p*)** | |
| Overall condition  (1–7 score) | D0 | | Score | 3.18 ± 0.98 | – | | 2.70 ± 0.82 | – | | 3.00 ± 0.47 | – | |
|  |  |  | Between group (*p*) | – | | | – | | | – | | |
|  | D28 | | Score | 3.91 ± 1.81 | <0.05 | | 3.70 ± 0.95 | <0.01 | | 2.90 ± 0.32 | - | |
|  |  |  | Between group (*p*) | – | | | – | | | – | | |
|  | **Source** | | | T | | | G | | | T × G | | |
|  | ***F* value** | | | 10.16 | | | 1.12 | | | 3.68 | | |
|  | ***p* value** | | | <0.01 | | | – | | | <0.05 | | |
| **Evaluation parameters (reference range)** |  | | | **Group 1 (*n*=11)** | | | **Group 2 (*n*=11)** | | | **Control (*n*=10)** | | |
|  |  |  |  | **Mean ± SD** | **Within group (*p*)** | | **Mean ± SD** | **Within group (*p*)** | | **Mean ± SD** | **Within group (*p*)** | |
| Overall QoL  (0–10 score) | D0 | | Score | 4.27 ± 2.28 | – | | 2.90 ± 1.10 | – | | 3.20 ± 0.92 | – | |
|  |  |  | Between group (*p*) | – | | | – | | | – | | |
|  | D28 | | Score | 5.36 ± 2.80 | <0.05 | | 4.50 ± 1.96 | <0.01 | | 3.10 ± 0.74 | – | |
|  |  |  | Between group (*p*) | <0.05 | | | – | | | – | | |
|  | **Source** | | | T | | | G | | | T × G | | |
|  | ***F* value** | | | 7.50 | | | 2.94 | | | 2.48 | | |
|  | ***p* value** | | | <0.05 | | | – | | | – | | |

*n* = number of animals; Group 1 = high-dose group; Group 2 = low-dose group; D = study days (D0 = baseline, D14 = day 14, D28 = day 28); T = time effect; G = group effect; Mean ± SD = mean and standard deviation; Within-group (p) = p-value for comparisons with baseline within each group (only p < 0.05 shown); Between-group (p) = p-value for comparisons between each treatment group and the control at the same time point (only p < 0.05 shown).

Scores for individual items (appetite, activity level, sociability, interaction with the owner, and overall condition) were rated on a 7-point Likert scale, and overall QoL was rated on a 0–10 scale, with higher scores indicating better outcomes.

**Supplementary Table 2.** Summary of vital signs and physical parameters in dogs with canine cognitive dysfunction following donepezil depot administration over a 4‑week study period

| **Parameter** | **Time** | **Group 1 (*n*=11)** | **Group 2 (*n*=10)** | **Control (*n*=10)** | ***p* value (T)** | ***p* value (G)** | ***p* value (T×G)** | **Reference range** |
| --- | --- | --- | --- | --- | --- | --- | --- | --- |
| BW (kg) | D0 | 3.77 ± 1.51 | 4.45 ± 1.41 | 6.89 ± 6.19 | – | – | – | – |
|  | D14 | 3.78 ± 1.47 | 4.43 ± 1.43 | 6.84 ± 6.21 |  |  |  |  |
|  | D28 | 3.70 ± 1.42 | 4.35 ± 1.47 | 6.98 ± 6.36 |  |  |  |  |
| BCS | D0 | 5.36 ± 1.21 | 4.36 ± 1.36 | 4.70 ± 1.16 | <0.01 | – | – | 1–9 |
|  | D14 | 4.82 ± 0.87 | 4.09 ± 1.22 | 4.60 ± 1.17 |  |  |  |  |
|  | D28 | 4.64 ± 1.29 | 4.10 ± 1.37 | 4.60 ± 1.17 |  |  |  |  |
| Temp (°C) | D0 | 38.80 ± 0.54 | 38.59 ± 0.40 | 38.72 ± 0.29 | – | – | – | 37.7–39.2 |
|  | D14 | 38.65 ± 0.62 | 38.74 ± 0.39 | 38.67 ± 0.29 |  |  |  |  |
|  | D28 | 38.58 ± 0.74 | 38.58 ± 0.31 | 38.62 ± 0.60 |  |  |  |  |
| HR (bpm) | D0 | 124.73 ± 15.56 | 115.55 ± 9.91 | 117.60 ± 15.23 | – | – | – | 90–140 |
|  | D14 | 125.73 ± 8.42 | 114.18 ± 13.04 | 118.30 ± 16.88 |  |  |  |  |
|  | D28 | 121.09 ± 17.61 | 115.90 ± 11.52 | 117.20 ± 13.79 |  |  |  |  |
| RR (/min) | D0 | 26.09 ± 7.69 | 28.82 ± 5.98 | 26.80 ± 6.32 | – | – | – | 18–34 |
|  | D14 | 26.27 ± 6.00 | 28.00 ± 5.74 | 27.30 ± 7.32 |  |  |  |  |
|  | D28 | 28.09 ± 8.44 | 28.20 ± 3.33 | 27.70 ± 6.93 |  |  |  |  |
| SBP (mmHg) | D0 | 129.82 ± 20.10 | 121.45 ± 8.54 | 125.00 ± 13.47 | – | <0.05 | – | 120–140 |
|  | D14 | 131.09 ± 15.63 | 116.00 ± 5.85 | 120.60 ± 11.25 |  |  |  |  |
|  | D28 | 137.27 ± 27.63 | 119.20 ± 8.66 | 120.50 ± 9.42 |  |  |  |  |

*n* = number of animals; Group 1 = high-dose group; Group 2 = low-dose group; D = study days (D0 = baseline, D14 = day 14, D28 = day 28); T = time effect; G = group effect; Mean ± SD = mean and standard deviation; BW = body weight; BCS = body condition score; Temp = rectal temperature; HR = heart rate; RR = respiratory rate; SBP = systolic blood pressure; p-values are presented only when <0.05.

**Supplementary Table 3.** Hematological parameters in dogs with canine cognitive dysfunction following donepezil depot administration over a 4‑week study period

| **Parameter** | **Time** | **Group 1 (*n*=11)** | **Group 2 (*n*=10)** | **Control (*n*=10)** | ***p* value (T)** | ***p* value (G)** | ***p* value (T×G)** | **Reference range** |
| --- | --- | --- | --- | --- | --- | --- | --- | --- |
| WBC (10^9^/L) | D0 | 12.06 ± 2.54 | 11.03 ± 6.08 | 12.44 ± 3.61 | – | – | – | 5.05–17.80 |
|  | D14 | 11.34 ± 2.84 | 10.84 ± 4.42 | 16.83 ± 14.49 |  |  |  |  |
|  | D28 | 11.39 ± 3.36 | 10.83 ± 4.46 | 14.02 ± 7.25 |  |  |  |  |
| LYM (K/μL) | D0 | 2.18 ± 1.37 | 1.86 ± 0.75 | 1.74 ± 0.37 | <0.05 | – | – | 1.05–5.10 |
|  | D14 | 2.50 ± 0.99 | 2.27 ± 1.22 | 2.38 ± 0.97 |  |  |  |  |
|  | D28 | 2.18 ± 1.15 | 2.16 ± 0.97 | 2.24 ± 0.75 |  |  |  |  |
| MONO (K/μL) | D0 | 1.24 ± 0.55 | 1.17 ± 0.73 | 1.15 ± 0.60 | – | – | – | 0.3–1.5 |
|  | D14 | 2.44 ± 3.61 | 1.26 ± 0.81 | 1.77 ± 2.34 |  |  |  |  |
|  | D28 | 1.22 ± 0.57 | 1.23 ± 0.84 | 1.43 ± 1.02 |  |  |  |  |
| EOS (K/μL) | D0 | 0.55 ± 0.44 | 0.41 ± 0.25 | 0.41 ± 0.26 | – | – | – | 0.06–1.23 |
|  | D14 | 0.52 ± 0.20 | 0.40 ± 0.24 | 0.34 ± 0.25 |  |  |  |  |
|  | D28 | 0.50 ± 0.26 | 0.51 ± 0.57 | 0.42 ± 0.25 |  |  |  |  |
| RBC (10^12^/L) | D0 | 7.16 ± 2.00 | 7.03 ± 1.07 | 7.27 ± 1.45 | <0.01 | – | – | 5.65–8.87 |
|  | D14 | 5.90 ± 0.90 | 6.16 ± 1.09 | 6.33 ± 2.36 |  |  |  |  |
|  | D28 | 6.18 ± 1.48 | 6.27 ± 0.88 | 6.70 ± 1.49 |  |  |  |  |
| HGB (g/dL) | D0 | 16.58 ± 4.08 | 16.23 ± 2.61 | 17.03 ± 2.70 | <0.01 | – | – | 13.1–20.5 |
|  | D14 | 13.81 ± 1.61 | 14.23 ± 2.12 | 14.49 ± 5.52 |  |  |  |  |
|  | D28 | 13.98 ± 3.18 | 14.40 ± 1.61 | 15.63 ± 2.91 |  |  |  |  |
| HCT (%) | D0 | 48.26 ± 12.44 | 47.64 ± 8.42 | 48.94 ± 8.87 | <0.01 | – | – | 37.3–61.7 |
|  | D14 | 39.98 ± 4.62 | 41.52 ± 5.57 | 42.19 ± 13.90 |  |  |  |  |
|  | D28 | 40.61 ± 9.48 | 41.86 ± 4.24 | 45.04 ± 8.01 |  |  |  |  |
| PLT (K/μL) | D0 | 449.91 ± 196.33 | 456.64 ± 173.77 | 488.10 ± 176.63 | – | – | – | 200–500 |
|  | D14 | 524.45 ± 221.30 | 543.27 ± 193.32 | 481.10 ± 250.32 |  |  |  |  |
|  | D28 | 468.36 ± 261.45 | 568.41 ± 199.89 | 528.20 ± 224.53 |  |  |  |  |

*n* = number of animals; Group 1 = high-dose group; Group 2 = low-dose group; D = study days (D0 = baseline, D14 = day 14, D28 = day 28); T = time effect; G = group effect; Mean ± SD = mean and standard deviation; WBC = white blood cell count; LYM = lymphocytes; MONO = monocytes; EOS = eosinophils; RBC = red blood cell count; HGB = hemoglobin; HCT = hematocrit; PLT = platelet count; p-values are presented only when <0.05.

**Supplementary Table 4.** Serum biochemical profiles in dogs with canine cognitive dysfunction following donepezil depot administration over a 4‑week study period

| **Parameter** | **Time** | **Group 1 (*n*=11)** | **Group 2 (*n*=10)** | **Control (*n*=10)** | ***p* value (T)** | ***p* value (G)** | ***p* value (T×G)** | **Reference range** |
| --- | --- | --- | --- | --- | --- | --- | --- | --- |
| ALT (U/L) | D0 | 73.00 ± 56.39 | 99.64 ± 55.00 | 97.60 ± 86.93 | – | – | – | 10–125 |
|  | D14 | 67.09 ± 66.61 | 115.91 ± 63.82 | 69.00 ± 25.99 |  |  |  |  |
|  | D28 | 66.09 ± 54.34 | 103.73 ± 64.47 | 59.00 ± 23.82 |  |  |  |  |
| ALP (U/L) | D0 | 98.00 ± 70.20 | 228.55 ± 270.65 | 217.30 ± 293.12 | – | – | – | 18–236 |
|  | D14 | 112.55 ± 79.22 | 231.91 ± 257.18 | 213.20 ± 282.13 |  |  |  |  |
|  | D28 | 102.73 ± 99.31 | 206.18 ± 170.91 | 193.60 ± 287.78 |  |  |  |  |
| GGT (U/L) | D0 | 4.00 ± 6.50 | 5.00 ± 3.22 | 7.80 ± 13.52 | – | – | – | 0–11 |
|  | D14 | 4.55 ± 6.23 | 4.64 ± 3.72 | 5.50 ± 5.25 |  |  |  |  |
|  | D28 | 3.55 ± 5.97 | 4.32 ± 2.81 | 2.20 ± 2.15 |  |  |  |  |
| TBIL (mg/dL) | D0 | 0.11 ± 0.01 | 0.11 ± 0.01 | 0.11 ± 0.01 | – | – | – | 0–1 |
|  | D14 | 0.10 ± 0.02 | 0.10 ± 0.00 | 0.10 ± 0.01 |  |  |  |  |
|  | D28 | 0.10 ± 0.00 | 0.10 ± 0.00 | 0.10 ± 0.01 |  |  |  |  |
| ALB (g/dL) | D0 | 2.55 ± 0.31 | 2.84 ± 0.38 | 2.62 ± 0.68 | <0.05 | - | <0.05 | 2.2–3.9 |
|  | D14 | 2.63 ± 0.29 | 2.78 ± 0.36 | 2.79 ± 0.71 |  |  |  |  |
|  | D28 | 2.62 ± 0.35 | 2.81 ± 0.42 | 2.96 ± 0.52 |  |  |  |  |
| GLOB (g/dL) | D0 | 4.57 ± 1.01 | 4.56 ± 0.93 | 3.98 ± 0.95 | – | – | – | 2.1–4.9 |
|  | D14 | 4.52 ± 0.65 | 4.57 ± 0.91 | 4.24 ± 0.78 |  |  |  |  |
|  | D28 | 4.46 ± 0.62 | 4.45± 0.90 | 4.34 ± 0.59 |  |  |  |  |
| TP (g/dL) | D0 | 7.12 ± 1.21 | 7.41 ± 0.77 | 6.56 ± 1.42 | – | – | – | 5.3–8.4 |
|  | D14 | 7.15 ± 0.79 | 7.36 ± 0.98 | 7.00 ± 1.25 |  |  |  |  |
|  | D28 | 7.08 ± 0.66 | 7.27 ± 0.87 | 7.27 ± 0.67 |  |  |  |  |
| CHOL (mg/dL) | D0 | 187.82 ± 75.81 | 232.73 ± 67.02 | 228.50 ± 88.32 | – | – | – | 100–330 |
|  | D14 | 204.73 ± 73.07 | 224.64 ± 68.97 | 243.60 ± 102.58 |  |  |  |  |
|  | D28 | 205.09 ± 84.39 | 225.14 ± 58.76 | 250.00 ± 117.80 |  |  |  |  |
| BUN (mg/dL) | D0 | 22.24 ± 9.66 | 27.65 ± 18.02 | 18.36 ± 9.47 | – | – | – | 7–29 |
|  | D14 | 21.15 ± 8.71 | 28.31 ± 17.84 | 19.36 ± 6.36 |  |  |  |  |
|  | D28 | 24.21 ± 16.96 | 29.30 ± 18.72 | 19.99 ± 5.42 |  |  |  |  |
| CREA (mg/dL) | D0 | 0.54 ± 0.30 | 0.75 ± 0.55 | 0.53 ± 0.26 | – | – | – | 0.3–1.5 |
|  | D14 | 0.51 ± 0.31 | 0.84 ± 0.54 | 0.57 ± 0.25 |  |  |  |  |
|  | D28 | 0.50 ± 0.25 | 0.83 ± 0.51 | 0.62 ± 0.23 |  |  |  |  |
| PHOS (mg/dL) | D0 | 4.12 ± 1.09 | 4.56 ± 1.12 | 3.90 ± 0.68 | – | – | – | 2–6 |
|  | D14 | 4.04 ± 0.49 | 4.93 ± 0.93 | 4.34 ± 0.93 |  |  |  |  |
|  | D28 | 4.04 ± 0.92 | 4.50 ± 0.66 | 4.61 ± 1.63 |  |  |  |  |
| LIPA (U/L) | D0 | 247.09 ± 256.65 | 188.27 ± 272.10 | 135.70 ± 111.47 | – | – | – | 25–400 |
|  | D14 | 100.00 ± 56.24 | 118.45 ± 74.51 | 131.80 ± 65.37 |  |  |  |  |
|  | D28 | 144.09 ± 132.70 | 149.59 ± 141.69 | 83.50 ± 29.04 |  |  |  |  |
| GLU (mg/dL) | D0 | 91.45 ± 22.37 | 99.82 ± 12.66 | 118.70 ± 66.52 | – | – | – | 74–146 |
|  | D14 | 90.09 ± 12.25 | 96.55 ± 13.34 | 107.90 ± 34.93 |  |  |  |  |
|  | D28 | 90.73 ± 20.39 | 103.64 ± 23.80 | 119.20 ± 89.37 |  |  |  |  |
| CA (mg/dL) | D0 | 10.18 ± 0.45 | 10.65 ± 0.92 | 10.12 ± 1.32 | – | – | – | 9.0–13.4 |
|  | D14 | 10.23 ± 0.79 | 10.52 ± 0.96 | 10.30 ± 1.70 |  |  |  |  |
|  | D28 | 9.57 ± 2.00 | 10.52 ± 1.11 | 10.79 ± 0.81 |  |  |  |  |
| K⁺ (mEq/L) | D0 | 5.12 ± 0.27 | 4.93 ± 0.79 | 4.81 ± 0.66 | – | – | – | 3.2–5.5 |
|  | D14 | 5.19 ± 0.58 | 5.02 ± 0.62 | 4.77 ± 0.46 |  |  |  |  |
|  | D28 | 5.47 ± 1.59 | 5.01 ± 0.68 | 4.98 ± 0.44 |  |  |  |  |
| Na⁺ (mEq/L) | D0 | 143.45 ± 5.59 | 141.55 ± 4.25 | 139.50 ± 5.68 | – | – | – | 138–158 |
|  | D14 | 141.91 ± 3.83 | 144.18 ± 3.22 | 144.80 ± 5.25 |  |  |  |  |
|  | D28 | 148.91 ± 13.26 | 144.45 ± 3.86 | 143.50 ± 4.77 |  |  |  |  |
| Cl⁻ (mEq/L) | D0 | 111.45 ± 2.84 | 110.27 ± 4.24 | 109.50 ± 3.47 | – | – | – | 107–122 |
|  | D14 | 109.45 ± 3.27 | 110.45 ± 2.94 | 110.70 ± 4.83 |  |  |  |  |
|  | D28 | 114.09 ± 11.15 | 110.86 ± 3.46 | 110.60 ± 4.14 |  |  |  |  |

*n* = number of animals; Group 1 = high-dose group; Group 2 = low-dose group; D = study days (D0 = baseline, D14 = day 14, D28 = day 28); T = time effect; G = group effect; Mean ± SD = mean and standard deviation; ALT = alanine aminotransferase; ALP = alkaline phosphatase; GGT = gamma-glutamyl transferase; TBIL = total bilirubin; ALB = albumin; GLOB = globulin; TP = total protein; CHOL = cholesterol; BUN = blood urea nitrogen; CREA = creatinine; PHOS = phosphorus; Ca = calcium; LIPA = lipase; GLU = glucose; K⁺ = potassium; Na⁺ = sodium; Cl⁻ = chloride; p-values are presented only when <0.05.

**Supplementary Table 5.** Frequency of adverse events observed in dogs with canine cognitive dysfunction following donepezil depot administration

| **Group** | **Day** | **Adverse event (*n*)** | **Description** |
| --- | --- | --- | --- |
| 1 | 14 | Lethargy (1), inner ear erythema (1) | Mild lethargy and erythema at the pinna; resolved spontaneously without intervention |
|  | 28 | Diarrhea (1), weight loss (1), lethargy (1), polyuria (1) | Diarrhea lasted ~4 days beginning 3 weeks post-treatment; slight body weight loss, mild lethargy, hyporexia, and polyuria also noted |
| 2 | 28 | Death (1)* | Died of acute pneumonia attributed to advanced age |

*n* = number of animals; Group 1 = high-dose group; Group 2 = low-dose group; D = study days (D14 = day 14, D28 = day 28); data are presented as the number of animals exhibiting each clinical sign during the 4 week study period.

*Assessed by the attending veterinarian as unrelated to drug administration.

**Supplementary Table 6.** Causality assessment of adverse events observed in dogs with canine cognitive dysfunction following donepezil depot administration

| **Evaluation** | **Time-point** | **Group 1 (*n*=11)** | **Group 2 (*n*=11)** | **Control (*n*=10)** | **Criteria Reference** |
| --- | --- | --- | --- | --- | --- |
| Occurrence | D14 | 1 | – | – | N/A |
|  | D28 | 2 | 2 | – |  |
| Intervention | D14 | 1 (I) | – | – | I: None  II: Symptomatic treatment  III: Hospitalization  IV: Drug discontinuation |
|  | D28 | 2 (I) | 2 (I) | – |  |
| Outcome | D14 | 1 (I) | – | – | I: Resolved  II: Improved  III: Unchanged  IV: Aggravated  V: Deceased  VI: Not followed |
|  | D28 | 2 (I) | 1 (I), 1 (V) | – |  |
| Causality | D14 | 1 (V) | – | – | I: Clearly related  II: Likely related  III: Possibly related  IV: Unlikely related  V: Not related  VI: Undeterminable |
|  | D28 | 2 (V) | 2 (V) | – |  |

*n* = number of animals; Group 1 = high-dose group; Group 2 = low-dose group; D = study days (D14 = day 14, D28 = day 28); “Occurrence” refers to the number of dogs that experienced at least one adverse event at the indicated time point; Numbers in parentheses indicate evaluation scores according to predefined criteria.
